# Carrying Capacity and Colonization Dynamics of *Curvibacter* in the *Hydra* Host Habitat

**DOI:** 10.3389/fmicb.2018.00443

**Published:** 2018-03-14

**Authors:** Tanita Wein, Tal Dagan, Sebastian Fraune, Thomas C. G. Bosch, Thorsten B. H. Reusch, Nils F. Hülter

**Affiliations:** ^1^Institute of Microbiology, Christian-Albrechts University of Kiel, Kiel, Germany; ^2^Institute of Zoology, Christian-Albrechts University of Kiel, Kiel, Germany; ^3^GEOMAR Helmholtz Centre for Ocean Research Kiel, Kiel, Germany

**Keywords:** *Curvibacter*, microbial ecology, host–microbe interactions, metaorganisms, *Hydra*

## Abstract

Most eukaryotic species are colonized by a microbial community – the microbiota – that is acquired during early life stages and is critical to host development and health. Much research has focused on the microbiota biodiversity during the host life, however, empirical data on the basic ecological principles that govern microbiota assembly is lacking. Here we quantify the contribution of colonizer order, arrival time and colonization history to microbiota assembly on a host. We established the freshwater polyp *Hydra vulgaris* and its dominant colonizer *Curvibacter* as a model system that enables the visualization and quantification of colonizer population size at the single cell resolution, *in vivo*, in real time. We estimate the carrying capacity of a single *Hydra* polyp as 2 × 10^5^ *Curvibacter* cells, which is robust among individuals and time. Colonization experiments reveal a clear priority effect of first colonizers that depends on arrival time and colonization history. First arriving colonizers achieve a numerical advantage over secondary colonizers within a short time lag of 24 h. Furthermore, colonizers primed for the *Hydra* habitat achieve a numerical advantage in the absence of a time lag. These results follow the theoretical expectations for any bacterial habitat with a finite carrying capacity. Thus, *Hydra* colonization and succession processes are largely determined by the habitat occupancy over time and *Curvibacter* colonization history. Our experiments provide empirical data on the basic steps of host-associated microbiota establishment – the colonization stage. The presented approach supplies a framework for studying habitat characteristics and colonization dynamics within the host–microbe setting.

## Introduction

Most eukaryotic organisms are colonized by a microbial community that is known to have diverse functions in health and development of their host (McFall-Ngai et al., 2013; Sommer and Bäckhed, 2013; Sommer et al., 2017). Yet, the rules governing the assembly of host-associated microbial communities are only beginning to be understood. A recent hypothesis posits that the microbiota constitutes an ecological community utilizing the host as an ecological habitat (Costello et al., 2012; Christian et al., 2015; Coyte et al., 2015). Under this view, a host can be described as a finite habitat for bacterial colonization. As such, it is characterized by multiple abiotic and biotic parameters with its carrying capacity as a prominent property. Habitat carrying capacity is defined as the maximum number of individuals that an environment, here the host, can sustain. The ecological carrying capacity has an evolutionary significance since population size is a well-known determinant of the population evolutionary dynamics (reviewed in Lanfear et al., 2014).

To study host colonization and carrying capacity in detail, we established a system to follow spatial and temporal dynamics of colonizer population size of the freshwater cnidarian *Hydra vulgaris* strain AEP (hereafter *Hydra*) and its dominant bacterial colonizer. Under constant laboratory conditions *Hydra* reproduces clonally; hence, individual polyps are assumed isogenic. *Hydra* stably maintains a highly specific microbiota, including three main families, whose composition under laboratory conditions is similar to that in nature (Fraune and Bosch, 2007; Fraune et al., 2015). The bacteria inhabit the outer layer of the ectodermal epithelial cells – termed glycocalyx (Fraune and Bosch, 2007; Fraune et al., 2015). The glycocalyx layer is considered as a physicochemical interface for *Hydra*–microbiota interactions (Schröder and Bosch, 2016). The microbial community residing in the glycocalyx has been observed to have an anti-fungal activity (Fraune et al., 2015), however, the role of specific community members remains unknown. The ability to generate germ-free polyps and to culture the microbiota members (Franzenburg et al., 2013; Fraune et al., 2015), makes it an ideal model system for the study of microbiota colonization dynamics over time. One bacterium, *Curvibacter* sp., was identified as the main colonizer of *Hydra*. The Gram-negative β-proteobacterium represents about 76% of the bacterial community stably associated with *Hydra* (Fraune et al., 2015).

The quantification of host carrying capacity provides an estimate for the total host-associated microbiota population size that in turn provides information about ecological and evolutionary trajectories of the inhabiting bacteria. Much less is known about the colonizer population size dynamics during the initial colonization phase from single colonizer cells to maximum population size (i.e., carrying capacity). If the host, here the *Hydra* polyp, is indeed viewed as a habitat, we expect the colonization dynamics to follow the same rules of establishment as in any ecological habitat [e.g., soil (Nemergut et al., 2006) or particles (Datta et al., 2016)]. Here we present a one host – one microbe system to study host colonization dynamics. Using genetically labeled *Curvibacter* strains, we characterize the colonizers’ population dynamics during initial colonization and quantify the *Hydra* habitat carrying capacity. The labeled *Curvibacter* strains are thus considered as a proxy for the total microbiota community (in which *Curvibacter* constitutes the majority). Furthermore, we test for priority effects in host colonization, and whether those are directly dependent on the time lag between competing colonizers.

## Materials and Methods

### Culturing of *Hydra* and Generation of Germ-Free Polyps

*Hydra vulgaris* (strain AEP) was routinely cultured according to standard procedures at 18°C (Lenhoff and Brown, 1970). Germ-free polyps were prepared by incubation in an antibiotic solution containing 50 μg ml^-1^ each of ampicillin, rifampicillin, streptomycin, and neomycin with daily exchange of the medium as previously described (Hemmrich et al., 2007). After 1 week of treatment, the polyps were transferred into antibiotic-free medium for recovery (1 week). The absence of bacteria was verified by plating on Reasoner’s 2A (R2A) medium agar plates. During antibiotic treatment and during re-colonization experiments, polyps were starved.

### Bacterial Strains, Plasmids, and Growth Conditions

The bacterial strains and plasmids used in this study are provided in Table S1, Supplementary File 1. *Curvibacter* sp. AEP1.3 was routinely grown in pure culture at 30°C with aeration in R2A medium or on R2A agar plates. When required, antibiotics were added to the medium at the following concentrations: Kanamycin (Km), 3 or 5 μg ml^-1^ and gentamicin (Gm), 2 μg ml^-1^. The *Curvibacter* sp. strains described in Table S1, Supplementary File 1 were constructed using standard molecular biology techniques including plasmid delivery via bi- and triparental mating using *E. coli* MFD*pir* (Ferrières et al., 2010) as plasmid donor. A mobilizable derivate of the gene targeting plasmid vector pGT41 (Kickstein et al., 2007) was used for the construction of the substitution/deletion mutation of the putative *flgC* gene involved in flagellar motility. A chromosomal mini-Tn*7* insertion (attTn7::miniTn*7(aacC1)*) was constructed using a transposon vector developed by Crepin et al. (2012). A detailed description of the plasmid and strain constructions is provided in the electronic Supplementary Material (Supplementary File 1).

### Colonization Assays

Germ-free *Hydra* polyps were divided in multiple pools with a defined number of animals in each group and kept in 50 ml *Hydra* medium (Lenhoff and Brown, 1970) throughout all experiments. The polyps were re-incubated with approximately 100 or 10^4^ cells per polyp for 24 h with cells from overnight cultures of either *Curvibacter* wild type or *Curvibacter* carrying either pRL153-GFP or pTW1-mCherry. After 24 h the medium was replaced in order to remove free cells. Every 24 h, three animals from one pool per time point were sampled, pooled and homogenized in an Eppendorf tube using a sterile pestle. Serial dilutions of the homogenate were plated on R2A agar plates or R2A agar plates supplemented with 5 μg ml^-1^ kanamycin to determine the CFUs per polyp and to distinguish between wild type and labeled (i.e., antibiotics resistant) populations. Experiments were carried out either for 168, 288 h or until a maximum of 312 h.

For experiments with *Hydra* polyps colonized with their natural resident microbiota, polyps were incubated with approximately 100 cells per polyp of *Curvibacter* carrying pRL153-GFP and treated as described above.

After acquiring the *Curvibacter* on-host population size (i.e., CFUs per polyp), non-linear least-squares minimization was used to fit a logistic growth model to the results and estimate the carrying capacity using the R package *growthcurver* (Sprouffske and Wagner, 2016).

### Fluorescence Microscopy of Colonized *Hydra* Polyps

*Hydra* polyps colonized by GFP- and mCherry-expressing *Curvibacter* cells were visualized with an epifluorescence microscope (Zeiss Axio Imager 2, Plan-Apochromat 63×/1.40 Oil DIC M27 objective). Single polyps were transferred on concave microscopy glass slides and subsequently cooled down on ice for 10 min prior to imaging.

### Priming Experiments

Two replicate populations of *Curvibacter* carrying plasmid pRL153-GFP were independently incubated on *Hydra* polyp pools for 168 h. After homogenization of the polyps, *Curvibacter* cells were carefully pelleted by centrifugation, recovered and serially diluted in *Hydra* medium. In parallel, two populations of *Curvibacter* wild type were incubated in R2A medium with daily transfers (1:100) for 168 h. Host-primed *Curvibacter* cells (obtained from the homogenized *Hydra* pools) and *Curvibacter* grown in R2A medium were used to initiate on-host-competition experiments. The experiments were conducted with approximately 100 cells per polyp of both *Curvibacter* strains. The number of both competitors per polyp was determined as described for the colonization assay.

## Results

### Establishing a Host–Microbe Model System: A Genetic Toolset for *Curvibacter*

**FIGURE 1:**
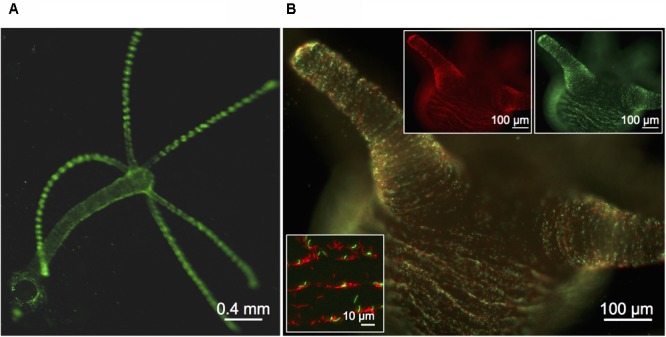
Visualization of fluorescently labeled *Curvibacter*. **(A,B)** Micrographs of a previously sterile polyp visualized 72 h post-infection with 6 × 10^3^ cells per polyp of *Curvibacter* carrying plasmid pRL153-GFP (green) or plasmid pTW1-mCherry (red). **(A)** Overview of a *Hydra* polyp densely colonized by GFP-expressing *Curvibacter*. **(B)** Side-view on the *Hydra* head region with mixed populations of GFP- and mCherry-expressing *Curvibacter*. White boxes: Fluorescence signals of the GFP- (top right) and mCherry-labeled population (top left). Close-up of *Curvibacter* cells densely settling in the proximity of the polyp’s folding molds (bottom left).

In order to visualize and follow bacterial growth and population dynamics on *Hydra* we equipped *Curvibacter* with the mobilizable RSF1010 derivative (IncQ) pRL153-GFP (carrying *nptII* with its original Tn*5* promoter and GFP expressed from the promoter *P_trc_*) (Tolonen et al., 2006). In biparental mating experiments with *E. coli* MFD*pir*, pRL153-GFP was effectively transferred to *Curvibacter* (frequency per recipients after 2 h: 5 × 10^-5^, *n* = 2, SEM = 2 × 10^-5^). For expression of the second fluorophore, we replaced GFP with mCherry (pTW1-mCherry). Infecting germ-free *Hydra* with marked *Curvibacter* lines enabled us to visualize *Curvibacter* populations on *Hydra* in real time at a single cell resolution (**Figures 1A,B**). We observed no impact of plasmid carriage on the fitness of *Curvibacter* compared to the plasmid-free wild type in pure culture (Table S3, Supplementary File 2). Consequently, the observed pattern of *Curvibacter* proliferation on *Hydra* cannot be explained by fluorophore expression or plasmid carriage.

Based on our observation that pRL153-GFP could be efficiently activated for conjugation, we developed a mobilizable derivate of the previously described ColE-derived gene targeting plasmid vector pGT41 (Kickstein et al., 2007; Overballe-Petersen et al., 2013; Hülter et al., 2017) that can be used for the generation of chromosomal deletion mutations in *Curvibacter* (see Supplementary File 1 for details).

### Visualization of *Curvibacter* Host Colonization

**FIGURE 2:**
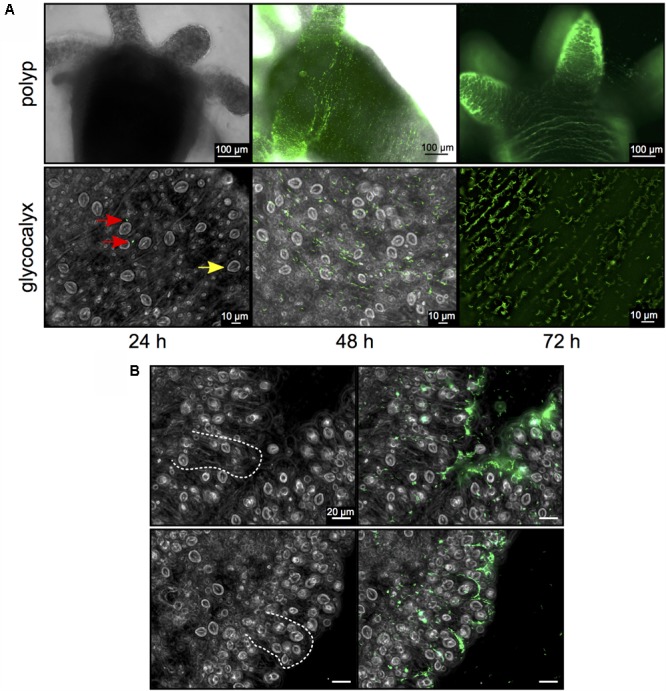
*Curvibacter* population size dynamics on *Hydra*. **(A)** Twenty-four hours post-infection, single *Curvibacter* cells (red arrows) are attached to the polyp (yellow arrow indicates a host nematocyst). Proliferation of *Curvibacter* leads to a dense layer of cells after 48 and 72 h. **(B)** Bright field micrographs of *Hydra* epithelium. Dashed lines indicate the shape of the epithelial battery cells filled with nematocyst in the tentacles of *Hydra* (left). *Curvibacter* carrying pRL153-GFP colonizing the *Hydra* epithelium along the shape of the epithelial muscle cells (right).

A pool of germ-free *Hydra* polyps was infected with an isogenic inoculum consisting of approximately 100 fluorophore-expressing *Curvibacter* cells per polyp. After 24 h polyps were washed and single *Curvibacter* cells were observed inhabiting *Hydra*. After 48 h patches of *Curvibacter* cells were observed on the *Hydra* epithelium and at 72 h the cells densely covered the polyp (**Figure 2A**). The *Curvibacter* cells pattern on the polyp revealed a gradual increase of the *Curvibacter* population size on *Hydra* over time. Furthermore, our results reveal that *Curvibacter* settles in neat lines that follow the structure of the epithelium, which is determined by the shape of individual epithelium muscle cells (Anton-Erxleben et al., 2009; **Figure 2B**). This distinct spatial pattern suggests that the continuous constriction movement of *Hydra* has an impact on the *Curvibacter* spatial distribution. After 72 h the highest density of *Curvibacter* was observed in the *Hydra* tentacles region, hence *Curvibacter* distribution on *Hydra* is not homogeneous rather it is biased toward specific body parts (**Figure 2A**), as recently shown (Augustin et al., 2017).

### Quantification of Host Carrying Capacity and Colonization Dynamics

**FIGURE 3:**
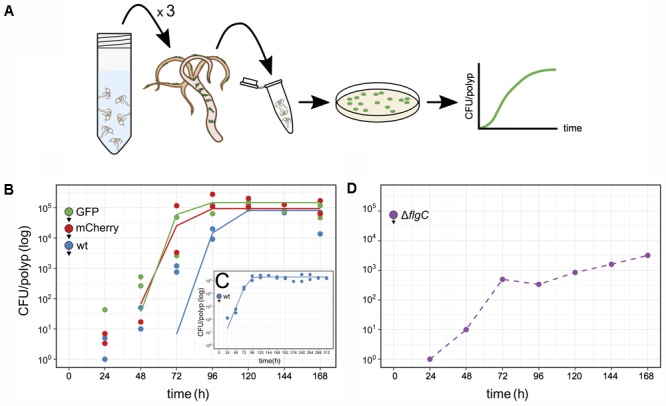
Quantification of *Curvibacter* population size on *Hydra*. **(A)** Schematic of the assay pipeline to follow colonization dynamics over time. Every 24 h, three polyps were sampled, homogenized, and the homogenate plated. **(B–D)** Population size of *Curvibacter* on *Hydra* over time. *Hydra* pools were incubated with 100 **(B,C)** or 10^4^ **(D)** cells per polyp (see section “Materials and Methods” for details). Filled circles with arrowheads indicate the bacterial strain and its arrival time on *Hydra*. **(B)** Population size of three differently labeled but otherwise isogenic populations [*n* = 2, wild type (wt), pRL153-GFP, and pTW1-mCherry] estimated from independent *Hydra* pools over time. Solid lines represent the best fit of a logistic growth model (wt *P*-value = 1.7 × 10^-4^, GFP *P*-value = 9.0 × 10^-6^, mCherry *P*-value = 5.4 × 10^-5^). **(C)** Long-time population size dynamic of *Curvibacter* on *Hydra* over 312 h shows logistic growth (*n* = 2, *P*-value = 4 × 10^-11^). **(D)** Population size of a motility impaired *Curvibacter flgC* deletion mutant over time (dashed line).

To further study *Curvibacter* colonization dynamics, we established a pipeline to quantify *Curvibacter* population size on *Hydra* over time (**Figure 3A**). We began by infecting two independent large germ-free *Hydra* polyp pools with *Curvibacter* (either wild type, or labeled). Batches of three polyps from each animal pool were sampled at consecutive time points and homogenized. The homogenate was plated on non-selective R2A plates. The number of colony forming units (CFUs) served as a direct estimate of the total bacterial population size on *Hydra* (**Figure 3A**). The quantification of CFUs per polyp for *Curvibacter* carrying pRL153-GFP or pTW1-mCherry was conducted by plating on non-selective R2A plates and R2A plates supplemented with kanamycin in parallel. No reduction in the plating efficiency of plasmid-carrying *Curvibacter* was observed. Hence, the CFU proxy was not affected by the marker presence. Furthermore, the plasmids pRL153-GFP and pTW1-mCherry were stable in *Curvibacter* under non-selective conditions. Only fluorescent colonies were found on non-selective R2A plates, hence the wild type CFU counts were not biased by the emergence of plasmid-free segregants.

Using the described pipeline, we followed the colonization dynamics of three lines, including the wild type, GFP, and mCherry lines in independent *Hydra* pools. At 24 h post-infection the *Curvibacter* population attached to *Hydra* was smaller than the inoculum size and reached a stable maximum after 96–120 h (**Figure 3B**). The gradual increase in CFU counts conforms to the increasing density of bacteria visually observed on *Hydra* over 72 h (**Figure 2A**). The wild type and the marked lines reached a similar stable maximum around 2 × 10^5^ cells per polyp 168 h post-infection. We note that the CFU per polyp dynamics were similar among the marked and non-marked lines, hence there is no difference in their relative fitness. Repeating the experiment for a longer time of 312 h showed that a maximum of about 2 × 10^5^ CFU per polyp was reached after 96 h and remained stable 312 h post-infection (**Figure 3C**). Conducting the colonization experiment with an inoculum size of 10^4^ cells per polyp showed that a similar stable maximum was rapidly reached 48–72 h post-infection (Supplementary Figure S1). In order to confirm that the source of the observed *Curvibacter* cells was indeed only from *Hydra*-associated bacteria we performed two control experiments. In the first we plated supernatant sampled from the polyp pool 48 h post-infection. No bacterial colonies could be observed in this experiment (less than two cells per milliliter), indicating that the *Curvibacter* colonies originate only from *Hydra* polyps. In addition, we incubated *Curvibacter* in *Hydra* medium supplemented with glucose and plated aliquots of the medium after 24 h. No colonies were observed on non-selective plates in this experiment, indicating that *Curvibacter* was not persisting in the *Hydra* medium. Consequently, we conclude that the colony counts in our pipeline truly reflect the abundance of *Hydra*-associated bacteria.

We further tested our approach to document colonization dynamics by investigating whether swimming motility plays a role in the early colonization process. *Curvibacter* is highly motile during early and mid-logarithmic growth phase and the presence of a full flagellum gene repertoire indicates that *Curvibacter* motility is flagellum-driven. For the purpose of this experiment we constructed a deletion mutant where a putative *flgC* gene was replaced by a marker cassette (strain Fm11; Table S1, Supplementary File 1). The *flgC* gene encodes a flagellar body rod protein and its absence is known to hamper bacterial motility (Shippy et al., 2014; Qin et al., 2016). Using our pipeline, we examined the host colonization dynamics of the Δ*flgC* strain (**Figure 3D**). Starting with an inoculum of about 100 cells per polyp revealed no colonies 24–168 h post-infection. Increasing the initial inoculum to 10^4^ cells per polyp improved the colonization success. About 500 cells per polyp were observed 72 h post-infection but only a maximum abundance of about 5 × 10^3^ cells was reached 168 h post-infection (**Figure 3D**). A comparison of these results to the wild type colonization experiment, which was done in parallel (e.g., **Figure 3B**), indicates that *Curvibacter* motility is an important factor for the colonization initiation and maintenance.

Overall, we find robust growth characteristics of *Curvibacter* in *Hydra* host colonization, namely, consistent growth kinetics of *Curvibacter* among hosts that are characterized by a simple logistic growth model (**Figures 3B,C**). We show that the *Hydra* carrying capacity amounts to about 2 × 10^5^ *Curvibacter* cells. This capacity is reached rapidly within 24–72 h, it is stable over long time, and it has robust characteristics among *Hydra* individuals.

### Colonizing Population Disturbance and Re-colonization

**FIGURE 4:**
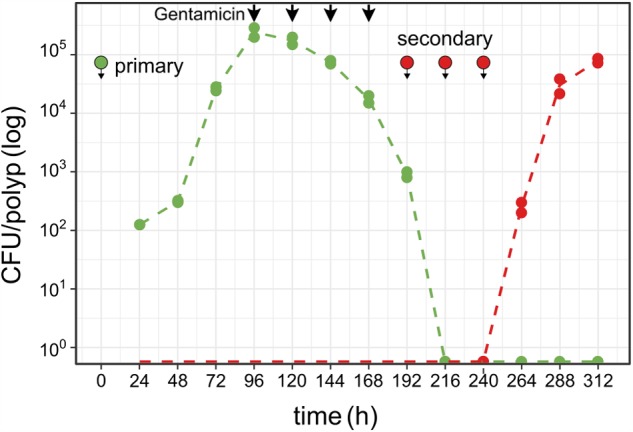
Colonizer population disturbance and re-colonization on *Hydra*. *Curvibacter* population size of first (green, pRL153-GFP) and second [red, *attTn7::miniTn7(aacC1)*] colonizer populations on *Hydra* (*n* = 2; dashed line represents the mean population size) during colonization and after colonization disruption by antibiotic treatment (black arrows, see section “Results” for details). Symbols indicate the time points of infection with *Curvibacter* populations.

To test the robustness of *Hydra* colonization and its carrying capacity we studied the colonization dynamics after an artificial disturbance of the colonizing *Curvibacter* population. *Curvibacter* is sensitive to gentamicin [adding 2 μg ml^-1^ gentamicin to a pure culture (10^8^ cells) resulted in no detectable CFU after 2 h of incubation]. Thus, we disturbed the host-inhabiting *Curvibacter* population by dosing the colonized *Hydra* polyps with gentamicin. In this experiment, we disturbed the *Curvibacter* population by treating the *Hydra* pool with a dose of 2 μg ml^-1^ gentamicin. The first treatment was applied at 96 h post-infection and was repeated every 24 h (without changing the medium) until no colonies could be observed. Overall, four gentamicin treatments were required to eliminate the total *Curvibacter* population (**Figure 4**). To test the *Hydra* carrying capacity robustness, we re-infected the *Hydra* pool with a gentamicin resistant *Curvibacter* (strain AEP1.3-Gm; Table S1, Supplementary File 1). The first re-infection was performed 24 h after the last gentamicin treatment (at 192 h) with the same inoculum size as before. After 24 h, no CFUs were detected; consequently we repeated the re-infection twice in time lags of 24 h until CFUs could be detected (at 264 h; **Figure 4**). Within 72 h the *Curvibacter* population size of the re-colonization phase reached the typical *Hydra* carrying capacity (∼2 × 10^5^). This experiment demonstrates that the *Hydra* carrying capacity is a robust characteristic of the host habitat that is independent of disturbance events. Additionally, we show that a decline in the existing colonizer population opens up ecological opportunities for new incoming colonizer populations.

### The Consequences of Host Carrying Capacity for Consecutive Colonization

Using the *Curvibacter*–*Hydra* model we tested the consequences of preemptive competition in the *Hydra* habitat during colonization. To quantify the impact of colonization order and time, we manipulated the time lag between two consecutive colonizer populations. Using our pipeline (**Figure 3A**), *Curvibacter* wild type served as the primary colonizer while *Curvibacter* carrying plasmid pRL153-GFP was used as the secondary colonizer. The secondary colonizer (100 cells per polyp) was added at different time lags of 24, 48, or 72 h post-primary infection. We evaluated the colonizer population size by plating simultaneously on non-selective and selective media. The growth characteristics and *Hydra* carrying capacity of both colonizers were estimated using a logistic growth model.

**FIGURE 5:**
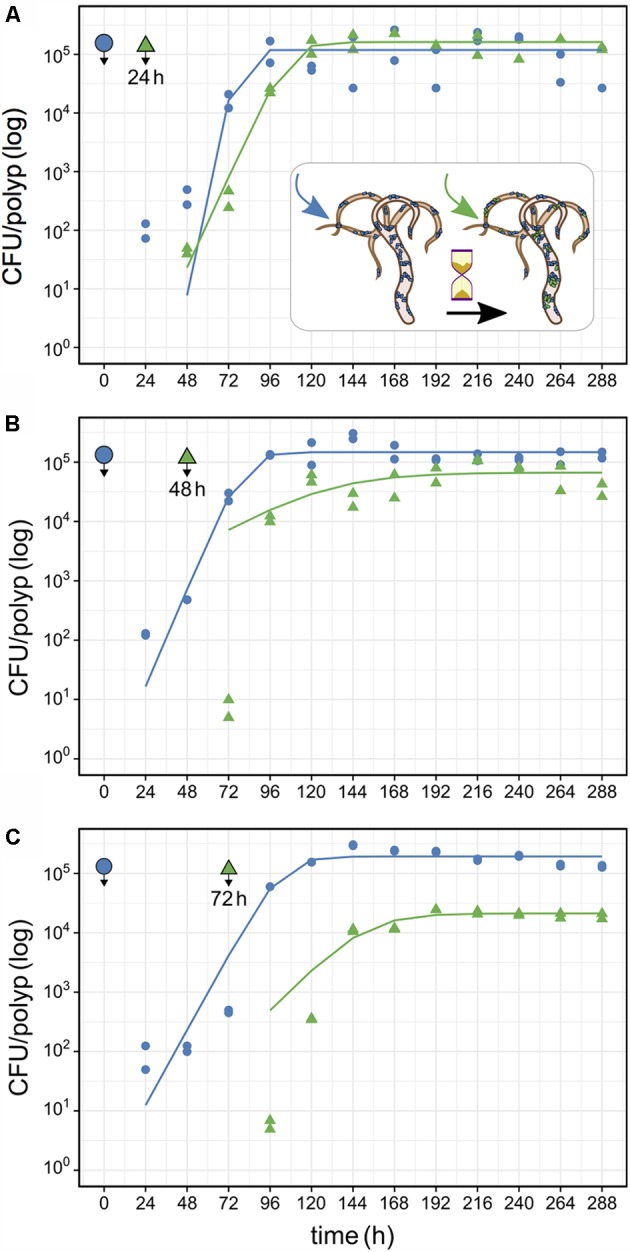
Population size of primary and secondary *Curvibacter* colonizers on *Hydra*. After infection with the primary colonizer (blue, wild type), the second colonizer (green, pRL153-GFP) was added after a time lag of **(A)** 24 h, **(B)** 48 h or **(C)** 72 h. All experiments were conducted with two biological replicates. Solid lines represent the best fit of a logistic growth model.

In the first time-lag experiment the secondary colonizer population was added 24 h post-primary infection. The secondary colonizer population size was slightly fluctuating over time but resulted in a similar density as the primary colonizer 288 h post-infection. The total *Hydra* carrying capacity in this experiment was similar to the observed for a single colonizer population (**Table 1** and **Figure 5**) and the carrying capacity of both colonizers remained stable over time (**Figure 5A**). Nevertheless, the secondary colonizer reached a slightly higher carrying capacity in comparison to the primary colonizer and constituted about 60% of the final colonizer population (**Table 1** and **Figure 5**). This suggests that the secondary colonizer had a slight advantage over the primary colonizer on *Hydra* under these conditions despite the later arrival.

**Table 1:** Carrying capacity (*K*) for consecutive *Curvibacter* colonization in the host habitat *Hydra* fitted to a logistic growth model.

Time lag	Colonizer	*K*	SE	Logistic growth model *P*-value	Total *K*	*K* ratio 2°/1°
24 h	1°	80,855	±7,243	1.4 × 10^-6^	207,309	∼60%
	2°	126,454	±18,270	0.0001		
48 h	1°	139,245	±16,730	1.6 × 10^-5^	202,242	∼30%
	2°	62,996	±11,847	0.0007		
72 h	1°	191,712	±19,912	4.9 × 10^-6^	214,222	∼10%
	2°	22,509	±1,643	9.4 × 10^-6^		

In the next experiment we applied a time lag of 48 h prior to infection of *Hydra* with the secondary colonizer. Shortly after the re-infection, secondary colonizers were observed inhabiting the host while their population size slightly fluctuated around a maximum of 2.5 × 10^4^ cells per polyp. Together, the two colonizers reached a stable population size after 96 h with a total population size (∼2 × 10^5^) similar to the estimates obtained from previous experiments. However, the secondary colonizer contributed to around 30% of the total colonizer population size only (**Table 1** and **Figure 5B**). At this time lag, the primary colonizer had a significant numerical advantage over the secondary colonizer that remained stable over time. Thus, in contrast to a time lag of 24 h, a time lag of 48 h between primary and secondary colonization results in different carrying capacity for the two colonizers (**Table 1** and **Figure 5B**).

The third experiment was performed with an extended time lag of 72 h between primary and secondary infection. Here, the secondary colonizer contributed about 10% to the total bacterial population size on *Hydra* (**Table 1**). This means that the carrying capacity for the consecutive colonizer was strongly reduced due to the high numerical advantage of the primary colonizer (**Table 1** and **Figure 5C**). Thus, with 48 and 72 h time lag the *Hydra* carrying capacity of the secondary colonizer is significantly lower in comparison to the primary colonizer, whereas the total carrying capacity is similar to the previous experiments and is stable over time (**Table 1** and **Figure 5C**).

To further test the consequences of host occupancy on the success of an incoming colonizer we performed a colonization experiment with *Hydra* individuals that were fully colonized by their natural microbiota. Our results revealed no CFU of the incoming *Curvibacter* colonizer over 168 h, hence the incoming *Curvibacter* population completely failed to colonize a previously populated *Hydra*.

Our results demonstrate that the time lag between consecutive colonization is a decisive determinant of their population size on *Hydra*. Since the *Hydra* carrying capacity is finite, the carrying capacity of an incoming colonizer largely depends on the *Hydra* habitat occupancy. Host occupancy is thus an important determinant of the establishment success of incoming colonizers.

### Colonization Dynamics of Primed and Naïve Colonizers

**FIGURE 6:**
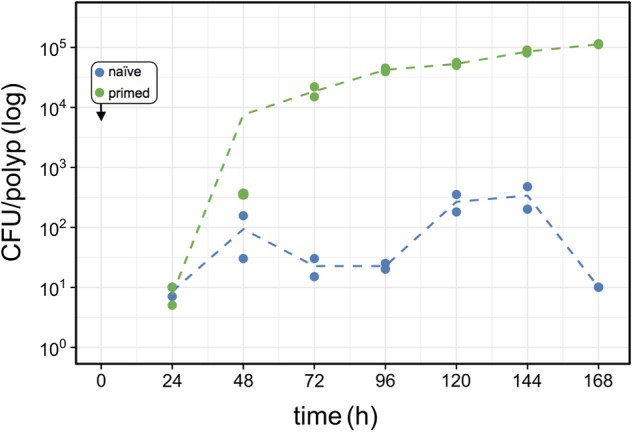
Effects of host-priming on *Curvibacter* fitness. Population size of primed *Curvibacter* carrying pRL153-GFP (green) and a naïve *Curvibacter* wild type (blue) on *Hydra* over time. Both *Curvibacte*r strains were added simultaneously to the *Hydra* pool (box) (*n* = 2; dashed line represents the mean population size).

To assess the source of advantage of the first colonizer we tested the effect of colonization history on the colonization success by performing a direct competition between primed and naïve colonizers. Primed colonizers (pRL153-GFP) were sampled from *Curvibacter* populations that were cultivated on *Hydra* prior to the experiment, while naïve colonizers (wild type) were sampled from *Curvibacter* populations maintained in liquid R2A medium (as in previous colonization experiments). A pool of germ-free *Hydra* polyps was inoculated with primed and naïve colonizers simultaneously at an equal density of approximately 100 cells per polyp. Using our pipeline, we documented changes in the primed and naïve colonizer population size by plating on non-selective and selective media. Our results reveal that 24 h post-infection both colonizers reach similar cell densities on the *Hydra* polyps. After 48 h the primed colonizer population size was slightly larger than that of the naïve colonizer, whereas at 72 h post-infection the primed colonizer largely outcompeted the naïve colonizer. Throughout the remaining experiment time, the naïve colonizer remained at low cell density of about 100 cells per polyp with a ratio of approximately 1:100 naïve to primed colonizer cells (**Figure 6**). To test whether this result was due to a general fitness benefit of the primed *Curvibacter* strain, we compared the fitness of the primed relative to the naïve strain in the absence of the host. A competition experiment between these two strains in liquid culture revealed no advantage of the primed strain after 24 or 48 h (Table S3, Supplementary File 2). Hence, the fitness advantage of the primed *Curvibacter* manifests itself only in the presence of the host. These experiments reveal that *Curvibacter* colonization history plays a major role in the host colonization success.

## Discussion

In our experiments we aimed to follow the total *Hydra* host colonization process over time and to test whether basic ecological concepts of habitat occupation (i.e., finite carrying capacity, robustness, and priority effects) hold true in a reductionist one host – one colonizer system. The ability to genetically modify *Curvibacter* enables the quantification of host colonization dynamics from single colonizer cells to growth-limited populations in real time. Our finding that IncQ plasmids are stably maintained in *Curvibacter* enables us to visualize *Curvibacter* colonization on *Hydra* and follow the emergence of colonization patterns over time. Our results confirm the hypothesis that the carrying capacity of *Hydra* as a habitat is finite, stable over time and has little variation among individual polyps (coefficient of variance between pairs of biological replicates did not exceed 23%). The carrying capacity we observed for *Curvibacter* is comparable to the total microbiota load on *Hydra* (Pietschke et al., 2017). Hence, our reductionist model appears realistic with regards to bacterial population size in the *Hydra* habitat. We demonstrate that the carrying capacity remains robust even after disturbance of the colonizer population. Furthermore, we show that the presence of colonizing bacteria in the host habitat decreases the carrying capacity of late colonizing bacteria. Thus, habitat occupancy is a determinant of colonizer carrying capacity, similarly to other ecological habitats. We show that the first arriving colonizer has a numerical advantage over late arrivals that is corresponding to the time lag to the second colonizer, a phenomenon that has been termed *priority effect* (reviewed in De Meester et al., 2016). The priority effect of first arriving *Curvibacter* is not only due to a numerical advantage as colonizers primed for *Hydra* have an advantage over naïve colonizers. This indicates that the priority effect in *Curvibacter* colonization of *Hydra* is enhanced by colonization history. Similarly to any other habitat, priority effects may be widespread in host–microbe interactions and might play an important role in host colonization. For example, a study comparing the colonization success of first and late arriving pathogenic *Borrelia burgdorferi* strains showed that the first arriving colonizer had a strong priority effect on the late arriving colonizer in a double host habitat comprising mice and ticks (Devevey et al., 2015). Nonetheless, the importance of colonization history in microbial community assembly was so far shown only for abiotic habitats (e.g., Gomez et al., 2016).

The colonization visualization with fluorescence microscopy revealed that *Curvibacter* cells are localized perpendicular to *Hydra’s* muscle cells, which suggests that the host constriction movements are the cause for *Curvibacter* spatial distribution on *Hydra*. The clear enrichment of *Curvibacter* in the tentacle area suggests that different *Hydra* body parts constitute distinct niches. It is thus likely that the host strongly influences bacterial cell localization and distribution in the host habitat. This observation is in agreement with a recent finding of a *Hydra* neuropeptide that is involved in patchy bacteria distribution on *Hydra* (Augustin et al., 2017). Additionally, the polyp movement appears to be an important determinant of bacterial population’s dynamics on the host and may strongly influence the colonization outcome. Similarly to *Hydra*, gut movements of the larval zebrafish were shown to significantly alter the colonization success of two colonizing populations (Wiles et al., 2016).

The robust characteristics of host habitat carrying capacity can be studied by a comparison of habitat characteristics among isogenic host individuals. In contrast to our results on *Hydra*, a study of bacteria residing in the gut of zebrafish larvae showed that the microbiota carrying capacity was variable among different individuals (Jemielita et al., 2014). This could be attributed to the strong gut motility leading to stochastic differences among individuals (Jemielita et al., 2014; Wiles et al., 2016). Furthermore, the host carrying capacity may be heterogeneous among different niches that correspond to sites or organs of the hosting organism. A study of plant microbiota of green snap beans (*Phaseolus vulgaris*) showed that leaf colonization by epiphytic bacteria occurs at multiple colonization sites having different carrying capacities (Remus-Emsermann et al., 2012). The patchiness in local carrying capacity could be attributed to a heterogeneous distribution of nutrients in the leaf habitat. Here, the total habitat carrying capacity of the green snap bean leaf is the sum of several local carrying capacities (Remus-Emsermann et al., 2012). The visualization of the unequal colonization of *Curvibacter* on *Hydra* may reveal a similar effect highlighting that a host can be comprised of different habitat niches.

Our results provide insights into the basic principles that govern host-associated bacterial colonization. As such, our data supports the idea that the microbiota is an ecological community utilizing the finite resources of a host habitat (Costello et al., 2012; Douglas and Werren, 2016). As it has been pointed out before, this does not rule out past or present co-evolution, as is the case in any other ecological community (Thompson and Cunningham, 2002; Thrall et al., 2007). The observed dominance and competitiveness of early colonizers constitute an extreme instance of ecological drift that is coupled with a strong population genetic bottleneck that is a consequence of the finite carrying capacity (Hu et al., 2006). An alternative mode of symbiont population bottleneck has been observed in the Squid–*Vibrio* symbiosis where the number of bacterial cells entering the light organ is limited and the community diversification occurs after the initial colonization (Wollenberg and Ruby, 2009; Sun et al., 2016).

A priority effect that is further enhanced by adaptation can lead to intraspecific genetic diversification of colonizers in distinct habitats (e.g., Gomez et al., 2016), where local adaptation and exclusion further promote the population divergence (Knope et al., 2012; Orsini et al., 2013). Using the *Hydra–Curvibacter* model we demonstrate the importance of colonization history, which may depend on specific host-mediated modifications of *Curvibacter* quorum-sensing signals (Pietschke et al., 2017), in host colonization and suggest ongoing adaptation that may lead to diversification and speciation. Thus, the phylogenetic relations of microbial populations reflect their ecological and evolutionary history. The application of ecological theory to explain host-associated microbiota establishment and colonization patterns is only just beginning. Our study provides first empirical data on the basic steps of host-associated microbiota establishment – the colonization stage in real time.

## Author Contributions

TW, TD, and NH designed the study. TW and NH performed the experiments. TW, TD, TR, and NH analyzed the data. SF and TB contributed *Hydra* polyps and wild type *Curvibacter* sp. AEP1.3 strain. TW, TD, SF, TB, TR, and NH wrote the manuscript.

## Conflict of Interest Statement

The authors declare that the research was conducted in the absence of any commercial or financial relationships that could be construed as a potential conflict of interest. The reviewer TM and handling Editor declared their shared affiliation.
