# Supplementary material for: Carrying Capacity and Colonization Dynamics of Curvibacter in the Hydra Host Habitat
Source: Front Microbiol. 2018 Mar 14;9:443. doi: 10.3389/fmicb.2018.00443 (PMC5861309; doi:10.3389/fmicb.2018.00443)
Supplement: Supplementary file 1 [file Presentation_1.pdf]

Supplementary Material

Carrying capacity and colonization dynamics of *Curvibacter* in the *Hydra* host habitat

Tanita Wein, Tal Dagan, Sebastian Fraune, Thomas C. G. Bosch, Thorsten B. H. Reusch, Nils F. Hülter*

* Correspondence: Nils F. Hülter: nhuelter@ifam.uni-kiel.de

Supplementary Materials and Methods

Bacterial strains and plasmids

The bacterial strains and plasmids used in this study are given in Table S1. Primers are listed in Supplementary Table S2. Plasmids were verified by restriction analysis and confirmed by sequencing. DNA fragments for cloning were amplified with Phusion proofreading polymerase (Thermo Fisher Scientific, Darmstadt, Germany). *Escherichia coli* strains were cultivated at 37°C in lysogeny broth (LB) with aeration. *E. coli* DH5α was used during plasmid constructions. Plasmids were moved into *E. coli* MFD*pir* (Ferrières et al., 2010), which served as the donor in mating experiments with *Curvibacter*, by chemical transformation. For cultivation of *E. coli* MFD*pir* the medium was amended with 0.1 mM diaminopimelic acid (DAP).

Construction of plasmid pTW1-mCherry

Plasmid vector pTW1 was constructed by PCR with plasmid pRL153-GFP as template and primers pRL153_fw and Ptrc_rv followed by intramolecular recircularization of the PCR fragment. We note that the insertion orientation of the P_{trc}::GFPmut3.1 fragment in the original plasmid pRL153-GFP is reversed as compared to its published description (Tolonen et al., 2006). To generate plasmid pTW1-mCherry, the open reading frame of mCherry was PCR-amplified with primer pair mChe_fw and mChe_rv and cloned blunt-end into the Eco53kI restriction site of pTW1. For plasmid delivery into *Curvibacter*, 0.5 ml of *E. coli* MFD*pir* and *Curvibacter* (both grown overnight at 30°C with aeration) were mixed, giving a donor-to-recipient ratio of approximately twenty to one. The bacteria were pelleted by centrifugation and resuspended in 0.5 ml R2A medium. The cell suspension was spotted onto non-selective R2A plates and incubated at 30°C for two hours. The cells were recovered in one ml R2A, serially diluted and plated on selective R2A plates supplemented with 5 µg ml^{-1} kanamycin.

Construction of *Curvibacter* sp. strain Fm11

The Δ*flgC* deletion in *Curvibacter* sp. Fm11 was constructed using the gene-targeting plasmid vector pGT42 for allelic replacement in a conjugation procedure. The ColE-based plasmid pGT42 (7920 bp) is a derivative of pGT41 (Kickstein et al., 2007) carrying a selective/counterselective marker cassette containing *nptII* (kanamycin resistance, Km^R) and *sacB* (susceptibility against sucrose, Suc^S) flanked by the unique restriction sites OliI and HpaI. Plasmid pGT42 was obtained by replacing the segment containing the phage f1-origin present in the backbone of the source plasmid vector pGT41 with the segment containing the *mobABC* genes and the origin of transfer region (*oriT*) from plasmid pRL153-GFP. For this, the backbone of pGT41 was amplified by inverse PCR with the primer pair pGT41_fw and pGT41_rv. Next, the resulting fragment (5863 bp) was phosphorylated and ligated to the *mobABC-oriT* containing PCR fragment generated with the primer pair mob-fw and mob-rv. The *flgC* gene was identified in the genome sequence of *Curvibacter* putative symbiont of *Hydra magnipapillata* (Csp_A00840; Chapman et al., 2010) using BLAST with proteobacterial *flgC* genes as input queries. We note that the genome sequence of *Curvibacter* putative symbiont of *Hydra magnipapillata* (BioProject: PRJEA39967) contains a second *flgC* gene (GenBank: Csp_D30210). A 795-bp segment upstream of the *flgC* gene was amplified from the *Curvibacter* chromosome using the primer pair flgC_up_fw and flgC_up_rv and inserted into the OliI site of the pGT42. The resulting plasmid pGT42-flgC-up was cut with HpaI and ligated to the PCR product of a segment downstream of *flgC* amplified with the primer pair flgC_down_fw and flgC_down_rv, giving plasmid pGT42-flgC-sub, which carried a Δ*flgC*::(*nptII sacB*) deletion allele embedded into its natural flanking regions. Delivery of the Δ*flgC*::(*nptII sacB*) deletion allele into *Curvibacter* gene was performed through conjugation with *E. coli* MFD*pir* carrying pGT42-flgC-sub as follows. Twenty ml overnight culture of *Curvibacter* grown at 30°C with aeration (approximately $4x10^8$ cells ml^{-1}) were mixed with 0.5 ml overnight culture of the *E. coli* donor strain. The mixture was pelleted by centrifugation, washed with R2A medium, resuspended in one ml of R2A medium, and spotted onto non-selective R2A plates supplemented with DAP (0.1 mM). After incubation for 18 h at 30°C, the bacterial lawn was recovered in R2A medium and entire mixture was plated on R2A plates supplemented with kanamycin (3 µg ml^{-1}) and incubated at 30°C. Resulting kanamycin resistant (and sucrose sensitive) colonies were verified by PCR using a combination of two sets of primer pairs, each with one primer located within the marker cassette and the corresponding reverse primer located outside of the cloned chromosomal segment present in the gene targeting plasmid (i.e., flgC-rj-1/sacB-P-sq and nptII-3-fw/flgC-rj-3) and additional Sanger sequencing.

Construction of *Curvibacter* sp. AEP1.3-Gm

A gentamicin resistant (Gm^R) mutant of *Curvibacter* (AEP1.3-Gm) was generated by insertion of a mini-Tn*7* transposon downstream of the conserved *glmS* gene (Csp_D281230; GenBank: FN543107.1) into the Tn*7*-specific *attTn7*-site, which is identical to the *attTn7*-site from *E. coli*. We used *E. coli* MFD*pir* carrying the mini-Tn*7* transposon vector pGP-Tn7-Gm (Crepin et al., 2012) and *E. coli* MFD*pir* carrying the transposase expression vector pTNS2 (Choi et al., 2005) as donor strains in a triparental mating procedure with *Curvibacter*. The mating protocol was identical to the procedure used for the generation of the Δ*flgC* mutant strain described above, with exception of the two *E. coli* donor strains carrying the mini-Tn*7* transposon vector and the transposase expression vector and plating of the recovered mating mixture on R2A plates supplemented with gentamicin (2 µg ml^{-1}). Gentamicin-resistant colonies were verified by PCR targeting the Tn*7*-insertion site with the primer pairs glmS-F/pTN7R and glmS-R/pTN7L and subsequent sequencing.

Fitness estimates

Standard Darwinian relative fitness (*w*) (Lenski et al., 1991) was estimated for host-primed and naïve *Curvibacter* (both carrying pRL153-GFP) in pairwise competition experiments with the naïve wildtype strain in R2A medium. Overnight cultures of the competitor strains were mixed in a ratio of 1:1 (500 µl of each). Cell densities of each competitor (CFU ml^{-1}) were determined by selective plating (R2A and R2A supplemented with 5 µg ml^{-1} kanamycin). The competition assays were diluted 100-fold in 1 ml fresh R2A medium and incubated for 24 h. The cultures were then diluted 100-fold in 1 ml fresh medium and incubated again for 24 hours as described above (i.e., 48 hours of competition in total). The ratio of the two competitors was determined after 24 and 48 hours by selective plating similar to the start-up of the experiment. Fitness estimates were determined as described in (Starikova et al., 2013).

Supplementary Table 1. Bacterial strains and plasmids

Strain or plasmid	***Relevant genotype and features***	***Reference***
Plasmids		
pRL153-GFP	P_{trc}::GFPmut3.1; *oriT-mob* region of RSF1010; *nptII* (Km^R)	(Tolonen et al., 2006)
pTW1	pRL153-GFP without GFPmut3.1 containing a Eco53kI site downstream of P_{trc}; *nptII* (Km^R)	this study
pTW1-mCherry	pTW1 containing P_{trc}::mCherry; *nptII* (Km^R)	this study
Strains		
Curvibacter sp.		
AEP1.3	wildtype	(Fraune et al., 2015)
AEP1.3/ pRL153-GFP	GFP, Km^R	this study
AEP1.3/ pTW1-mCherry	mCherry, Km^R	this study
Fm11	AEP1.3: Δ*flgC*[#]::(*sacB nptII*); Km^R	this study
AEP1.3-Gm	AEP1.3: *attTn7::miniTn7(aacC1)*; Gm^R	this study

[#] *flgC*: Csp_A00840; GenBank: FN543104.1 (*Curvibacter* putative symbiont of *Hydra magnipapillata* scaffold HmaUn_WGA69518_1). Km^R, kanamycin resistance; Gm^R, gentamicin resistance.

Supplementary Table 2. Oligonucleotides used in this study

Name	DNA sequence (5' to 3')
pRL153-fw	CTCAGCGACTCGACCGGTTCGCTGG
Ptrc-rv	CTCTAATTTCTCCTCTTTAATTCTAGG
Ptrc-seq-fw	CTCCTGCTCCGGTTTTCCAT
Ptrc-seq-rv	CTGGCCGAGGAGATTAACCC
mChe-fw	ATGGTGAGCAAGGGCGAGGAG
mChe-rv	**TTA**CTTGTACAGCTCGTCCATGC
pGT41-fw	CTCACTATAGGGCGAATTGG
pGT41-rv	CTCTATTCAGGCGTAGCAACCAG
mob-fw	CTGGAGCGCTTTTAGCCGCTTTAG
mob-rv	CATGCCTGATAGTTCTTCGGGC
flgC-up-fw	**G**TAAGCCCAGACACTCACCGGC
flgC-up-rv	**A**TCTA**G**ACCCTCTCAAAGGC
flgC-down-fw	C**TTC**TAGACGGCAGGGTTACC
flgC-down-rv	**AAC**TTGACGGCTCTGCGGCATC
flgC-rj-1	TGGGCGGCAATGGAAAAATC
flgC-rj-3	GAGCATTTGATGTCCGCCTG
sacB-P-sq	CAGCAGTGCGGTAGTAAAGG
nptII-3-fw	AAGCGAAACATCGCATCGAG
glmS-F	AAGATCGAGAGTGGCGAAGG
glmS-R	TGTGGCTGTGCTTGATCAGT
pTN7R	CACAGCATAACTGGACTGATTTC
pTN7L	ATTAGCTTACGACGCTACACCC

Bold letters indicate nucleotide exchanges introduced by the primer.

References

Chapman, J. A., Kirkness, E. F., Simakov, O., Hampson, S. E., Mitros, T., Weinmaier, T., et al. (2010). The dynamic genome of *Hydra*. *Nature* 464, 592–596. doi:10.1038/nature08830.

Choi, K.-H., Gaynor, J. B., White, K. G., Lopez, C., Bosio, C. M., Karkhoff-Schweizer, R. R., et al. (2005). A Tn*7*-based broad-range bacterial cloning and expression system. *Nat. Meth.* 2, 443–448. doi:10.1038/nmeth765.

Crepin, S., Harel, J., and Dozois, C. M. (2012). Chromosomal complementation using Tn*7* transposon vectors in *Enterobacteriaceae*. *Appl. Envir. Micro.* 78, 6001–6008. doi:10.1128/AEM.00986-12.

Ferrières, L., Hémery, G., Nham, T., Guérout, A.-M., Mazel, D., Beloin, C., et al. (2010). Silent mischief: bacteriophage Mu insertions contaminate products of *Escherichia coli* random mutagenesis performed using suicidal transposon delivery plasmids mobilized by broad-host-range RP4 conjugative machinery. *J. Bacteriol.* 192, 6418–6427. doi:10.1128/JB.00621-10.

Fraune, S., Anton-Erxleben, F., Augustin, R., Franzenburg, S., Knop, M., Schröder, K., et al. (2015). Bacteria–bacteria interactions within the microbiota of the ancestral metazoan *Hydra* contribute to fungal resistance. *ISME J.* 9, 1543–1556. doi:10.1038/ismej.2014.239.

Kickstein, E., Harms, K., and Wackernagel, W. (2007). Deletions of *recBCD* or *recD* influence genetic transformation differently and are lethal together with a *recJ* deletion in *Acinetobacter baylyi. Microbiology* 153, 2259–2270. doi:10.1099/mic.0.2007/005256-0.

Lenski, R. E., Rose, M. R., Simpson, S. C., and Tadler, S. C. (1991). Long-term experimental evolution in *Escherichia coli*. I. adaptation and divergence during 2,000 generations. *Amer. Nat.* 138, 1315–1341. doi:10.1086/285289.

Starikova, I., Al-Haroni, M., Werner, G., Roberts, A. P., Sorum, V., Nielsen, K. M., et al. (2013). Fitness costs of various mobile genetic elements in *Enterococcus faecium* and *Enterococcus faecalis*. *J. Antimicrob. Chemother.* 68, 2755–2765. doi:10.1093/jac/dkt270.

Tolonen, A. C., Liszt, G. B., and Hess, W. R. (2006). Genetic manipulation of *Prochlorococcus* strain MIT9313: Green fluorescent protein expression from an RSF1010 plasmid and Tn5 transposition. *Appl. Envir. Micro.* 72, 7607–7613. doi:10.1128/AEM.02034-06.
