# Supplementary material for: Carrying Capacity and Colonization Dynamics of Curvibacter in the Hydra Host Habitat
Source: Front Microbiol. 2018 Mar 14;9:443. doi: 10.3389/fmicb.2018.00443 (PMC5861309; doi:10.3389/fmicb.2018.00443)
Supplement: Supplementary file 2 [file Presentation_2.PDF]

Supplementary Material

Carrying capacity and colonization dynamics of *Curvibacter* in the *Hydra* host habitat

Tanita Wein, Tal Dagan, Sebastian Fraune, Thomas C. G. Bosch, Thorsten B. H. Reusch, Nils F. Hülter*

* Correspondence: Nils F. Hülter: nhuelter@ifam.uni-kiel.de

Supplementary Figure1 and Supplementary Table 3

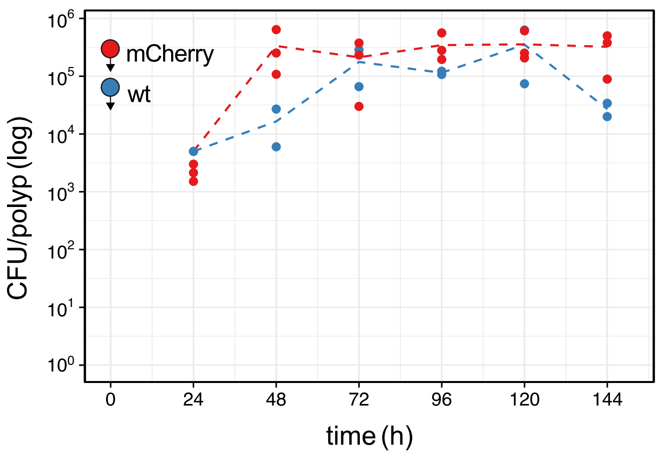

Supplementary Figure 1. Colonization and proliferation of *Curvibacter* on *Hydra* with high infection density. Germ-free *Hydra* polyps were infected with approximately 10^4 cells per polyp of either *Curvibacter* wild-type or carrying the plasmid pTW1-mCherry. Polyps got immediately colonized and reached the environmental capacity already after 48 h of incubation (n=2, dashed line represent the mean population size).

Supplementary Table 3. Cell titers (CFU/ml) of serial passage experiments with plasmid-carrying (pRL153-GFP) *Curvibacter* competed versus its plasmid-free wildtype (wt) counterpart and relative fitness[a] (*w*) of the naïve, plasmid-carrying (A) and the *Hydra*-primed[b], plasmid-carrying (B) *Curvibacter*.

	0 hours (start)		24 hours (1st passage)				48 hours (2nd passage)			
n	pRL153-GFP ($\times 10^6$)	wt ($\times 10^6$)	pRL153-GFP ($\times 10^8$)	wt ($\times 10^8$)	*P*-value[c]	$w_{pRL153\text{-}GFP}$	pRL153-GFP ($\times 10^8$)	wt ($\times 10^8$)	*P*-value[c]	$w_{pRL153\text{-}GFP}$
(A) Naïve, plasmid-carrying *Curvibacter versus* naïve *Curvibacter* wt										
1	1.2	0.8	1.94	4.36	0.125	0.81	1.84	3.48	0.125	1.04
2	0.78	1.1	2.44	3.62		0.99	2.16	3.64		0.97
3	2.9	3.2	4.28	5.02		0.99	4.52	5.98		0.97
4	3.3	2.7	2.74	3.30		0.92	3.98	4.44		1.02
(B) *Hydra*-primed, plasmid-carrying *Curvibacter versus* naïve *Curvibacter* wt										
1	0.46	0.52	1.40	3.04	0.125	0.90	1.86	2.84	0.097	1.08
2	0.64	0.88	2.36	2.94		1.02	2.52	2.92		1.02
3	2.3	1.7	4.28	4.62		0.93	4.50	5.48		0.97
4	2.4	3.0	3.60	4.02		1.02	4.10	4.20		1.02

[a] The ratio of the Malthusian parameters of the plasmid-carrying strain to that of its wt counterpart. A relative fitness close or equal to w=1.0 denotes no apparent difference in relative fitness (i.e., no cost of plasmid carriage).
[b] *Curvibacter* that had been cultivated on *Hydra* prior to the experiment for 168 h (i.e., priming).
[c] Wilcoxon rank-sum test used to test for differences in the final CFU/ml of the plasmid-carrying and the wt strain after 24 hours and 48 hours.
